# Supplementary figures and images for: Colon stroma mediates an inflammation-driven fibroblastic response controlling matrix remodeling and healing
Source: PLoS Biol. 2022 Jan 27;20(1):e3001532. doi: 10.1371/journal.pbio.3001532 (PMC8824371; doi:10.1371/journal.pbio.3001532)

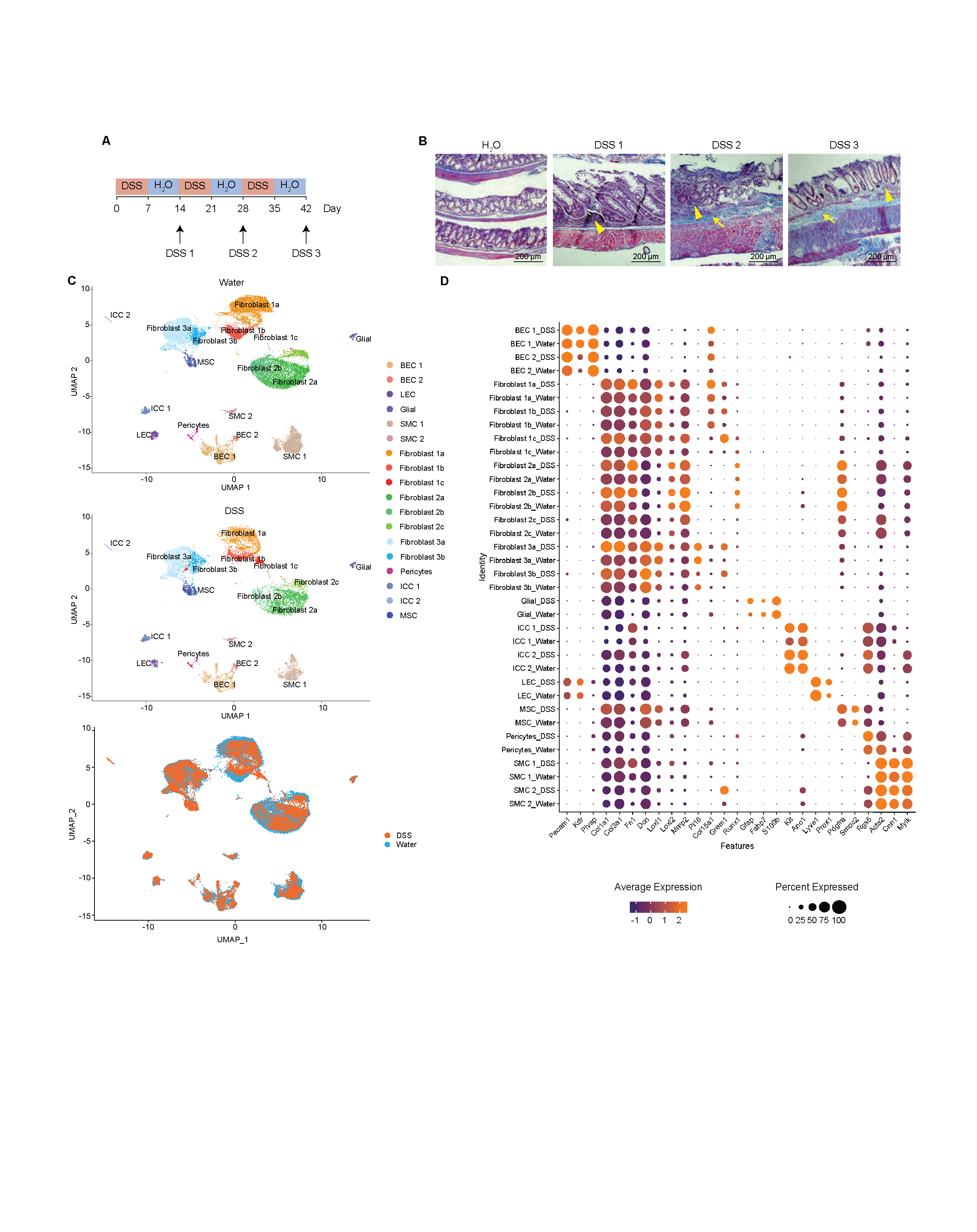

Supplement: S1 Fig — (A) Chronic DSS murine model: Mice were fed 3 iterative cycles of 2.5% DSS for 7 days followed by water for 7 days. (B) Masson’s trichrome staining of colons from water- and chronic DSS-fed mice after 1 round (day 14), 2 rounds (day 28), and 3 rounds (day 42) of DSS. Collagen accumulation in blue, as demarcated by yellow arrows. Leukocyte infiltrates, as demarcated by yellow arrowheads. Scale bar, 200 μm. n = 2, representative of 2 experiments. (C) Single-cell atlas of the murine colonic stroma. UMAP of stroma cells (dots) colored by cell type assignment from water (top) or DSS (middle) samples and combined embedding of both conditions (bottom panel). (D) Expression of lineage-specific marker genes across cell type subsets. Color represents average expression of marker gene within clusters; diameter represents percentage expression of marker gene within cluster. BEC, blood endothelial cell; DSS, dextran sulfate sodium; ICC, interstitial cell of Cajal; LEC, lymphatic endothelial cell; MSC, mesenchymal stem cell; SMC, smooth muscle cell; UMAP, uniform manifold approximation and projection. (TIF) [file pbio.3001532.s001.tif]

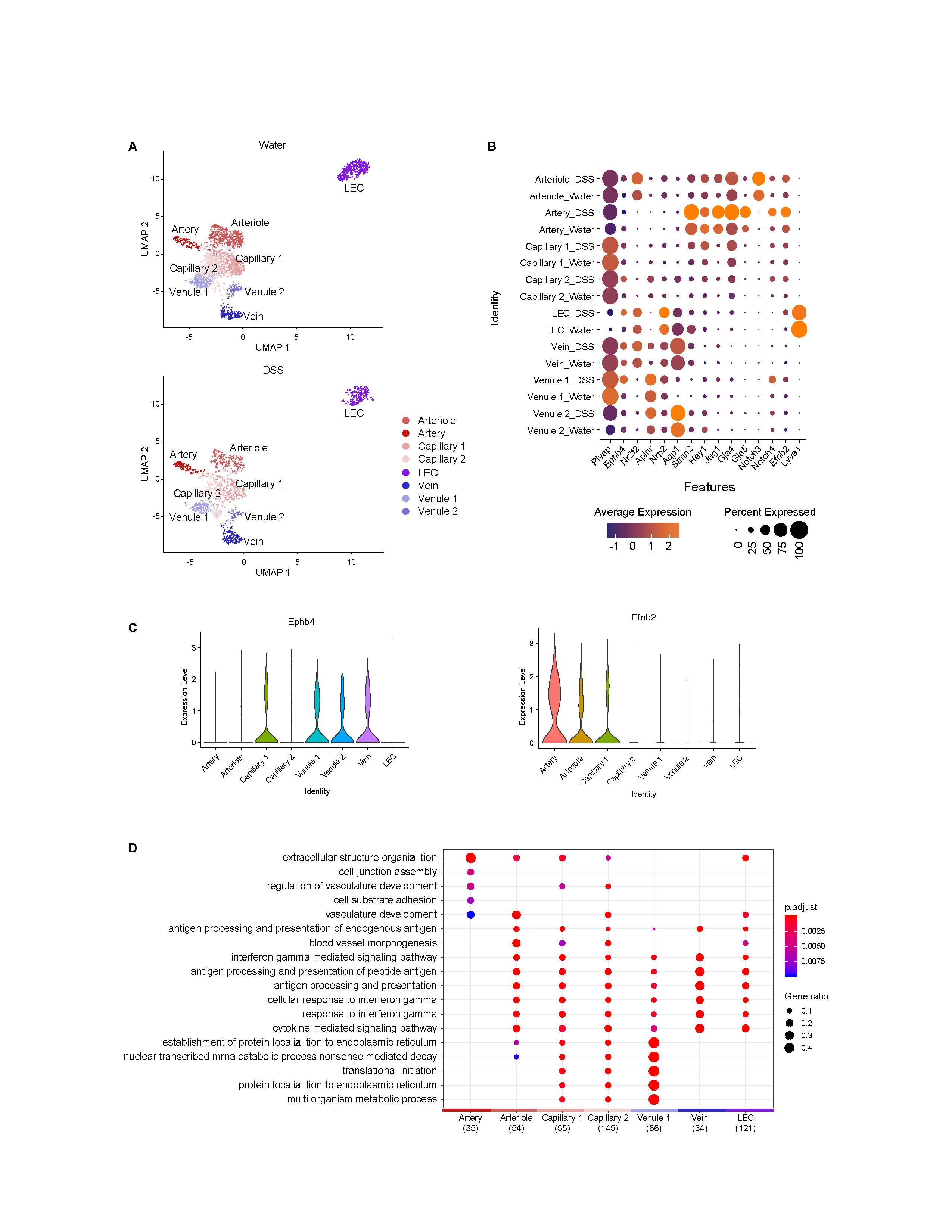

Supplement: S2 Fig — (A) Single-cell atlas of colon fibroblasts UMAP of endothelial cell (dots) profiles (Methods) colored by cell type assignment from water (top) or DSS (bottom) samples. (B) Expression of canonical markers across endothelial cell clusters. Color represents average expression of marker gene within clusters; diameter represents percentage expression of marker gene within cluster. (C) Violin plots of Ephb4 and Efnb2 expression level across endothelial cell clusters. Normalized gene expression levels are plotted on the y-axis. (D) GO enrichment of DEGs for each endothelial cell cluster between water- and DSS-treated samples. Color represents adjusted p-value of GO enrichment annotation for each endothelial cell cluster; diameter represents gene ratio for each endothelial cell cluster. DEG, differentially expressed gene; DSS, dextran sulfate sodium; GO, gene ontology; LEC, lymphatic endothelial cell; UMAP, uniform manifold approximation and projection. (TIF) [file pbio.3001532.s002.tif]

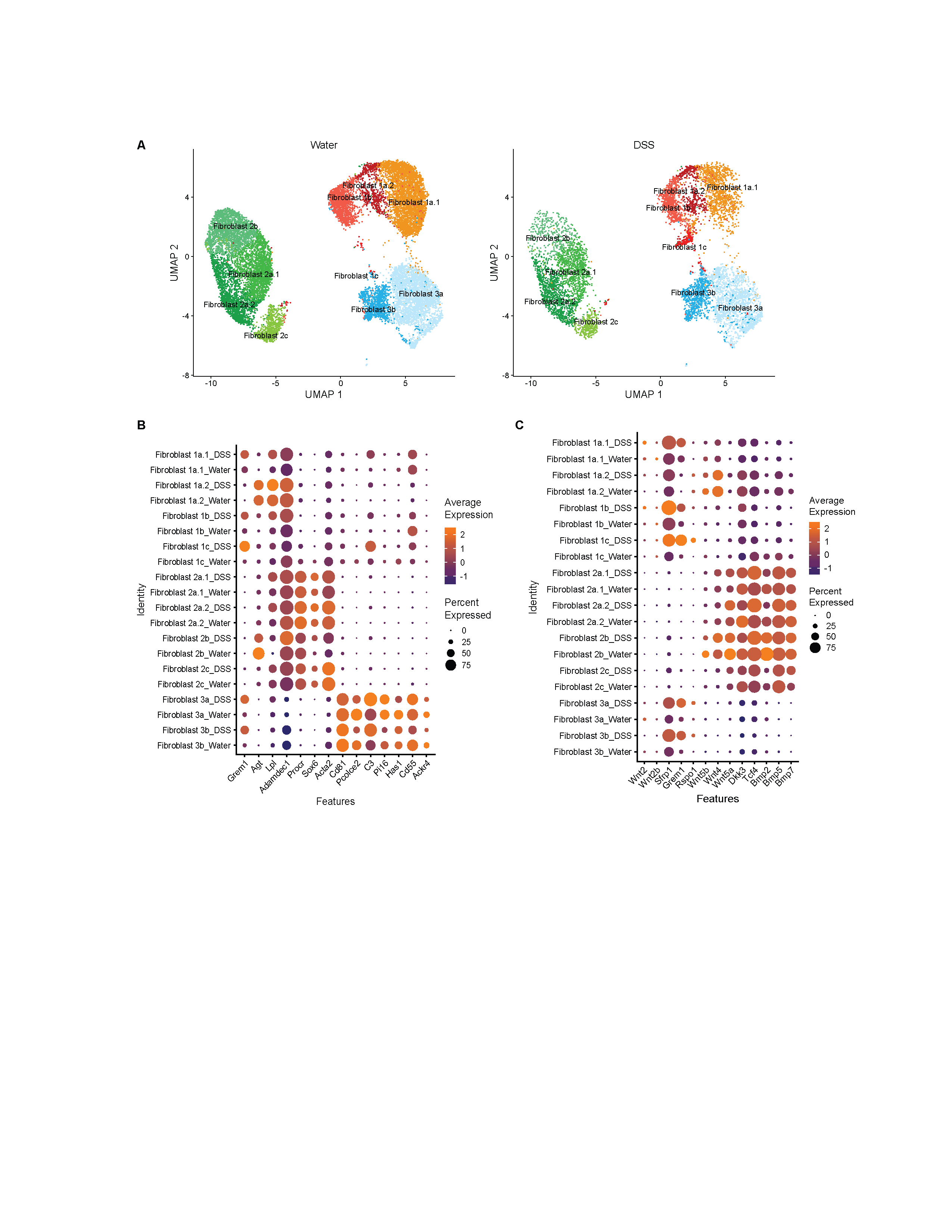

Supplement: S3 Fig — (A) Single-cell atlas of colon fibroblasts UMAP of fibroblast (dots) profiles (Methods) colored by cell type assignment from water (left) or DSS (right) samples. (B) Expression of canonical and newly characterized markers across fibroblast clusters. Color represents average expression of marker gene within clusters; diameter represents percentage expression of marker gene within cluster. (C) Expression of genes involved in maintaining colon crypt architecture. Color represents average expression of marker gene within clusters; diameter represents percentage expression of marker gene within cluster. DSS, dextran sulfate sodium; UMAP, uniform manifold approximation and projection. (TIF) [file pbio.3001532.s003.tif]

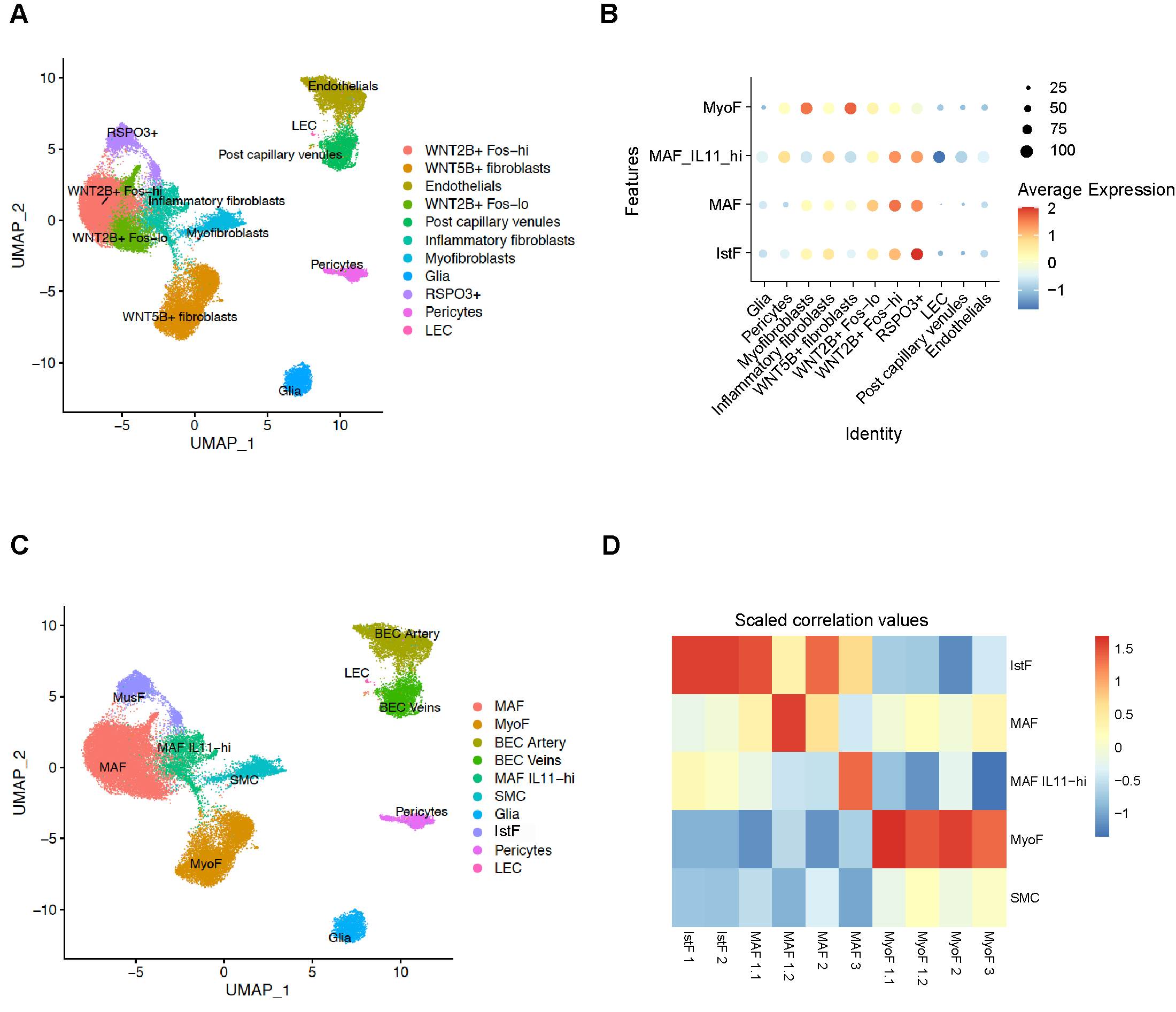

Supplement: S4 Fig — (A) UMAP of combined analysis and clustering of stromal cell subsets identified in [21] and [16]. Cell clusters obtained in the combined analysis were annotated based on the cell annotations defined by Smillie colleagues, using the most frequently present broader cell type annotation to annotate each cluster. (B) Dotplot of gene set module scores of genes defined to be highly specific to broader level mouse fibroblast subsets (x-axis) computed for each redefined human fibroblast cell subsets from joint clustering analysis. Only specific marker gene lists with AUC >0.65 were considered for computing gene set module score. Mouse fibroblast cell subsets were redefined as broader level subsets: IstF, MAF, and MyoF. MAF3 cell subset from mouse colon data was renamed to MAF IL11-hi. (C) UMAP of combined analysis and clustering of stromal cell subsets identified in Smilie and colleagues and Kinchen and colleagues with redefined cell type annotations aligned with broader mouse fibroblast cell type annotations. (D) Spearman correlation estimates of pseudobulk gene expression levels for each fibroblast cell subset identified in our mouse colon stroma atlas compared to pseudobulk expression levels of orthologous genes in each fibroblast cell subsets identified in a combined analysis of stromal cell subsets from Smilie and colleagues and Kinchen and colleagues. Correlation estimates were scaled for each column. AUC, area under the curve; BEC, blood endothelial cell; IstF, interstitial fibroblast; LEC, lymphatic endothelial cell; MAF, mucosa-associated fibroblast; MyoF, myofibroblast; SMC, smooth muscle cell; UMAP, uniform manifold approximation and projection. (TIF) [file pbio.3001532.s004.tif]

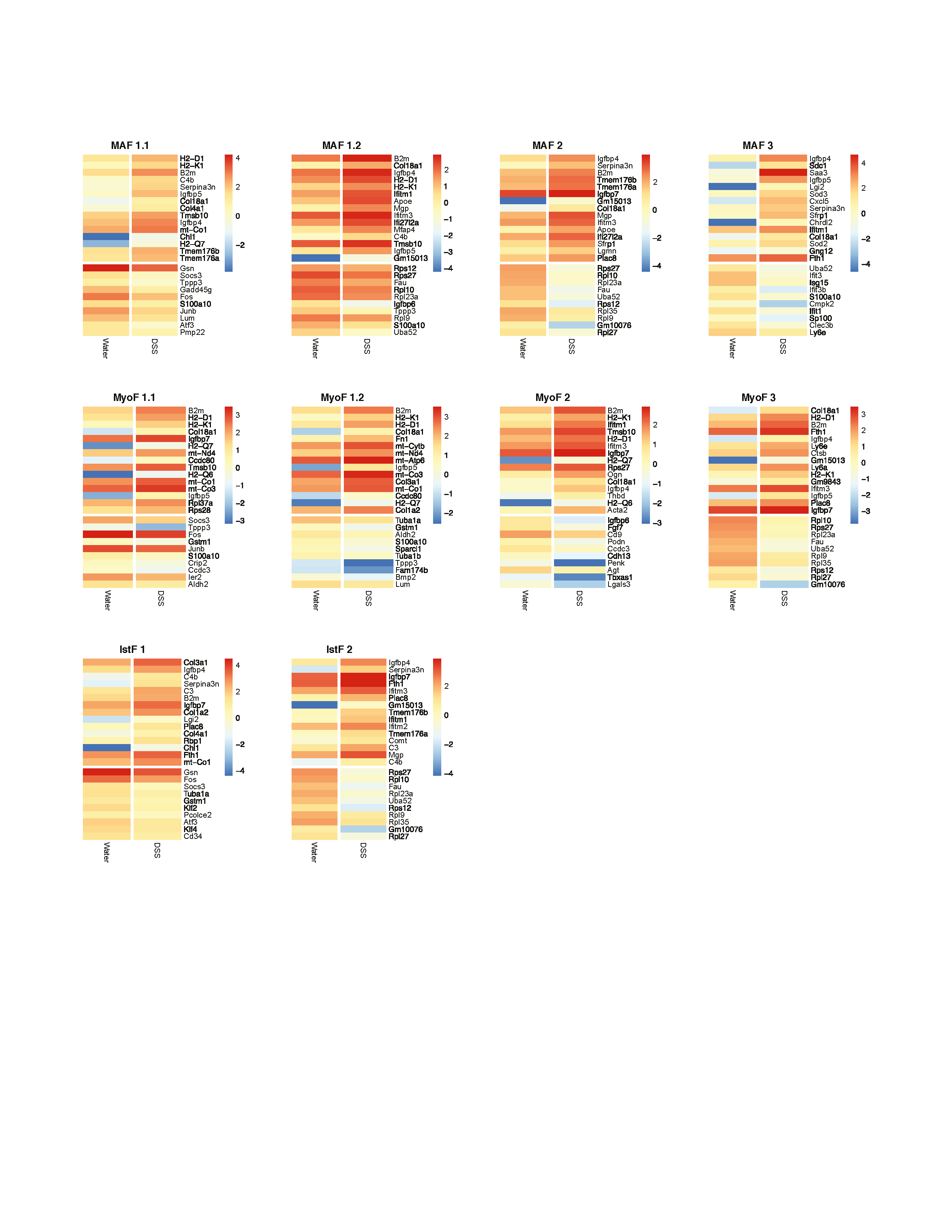

Supplement: S5 Fig — Heatmaps of select DEGs between water- and DSS-treated samples for all fibroblast clusters. Color represents normalized gene expression. Significant DEGs had FDR <0.05 using MAST (see Methods). DEG, differentially expressed gene; DSS, dextran sulfate sodium; FDR, false discovery rate; IstF, interstitial fibroblast; MAF, mucosa-associated fibroblast; MyoF, myofibroblast. (TIF) [file pbio.3001532.s005.tif]

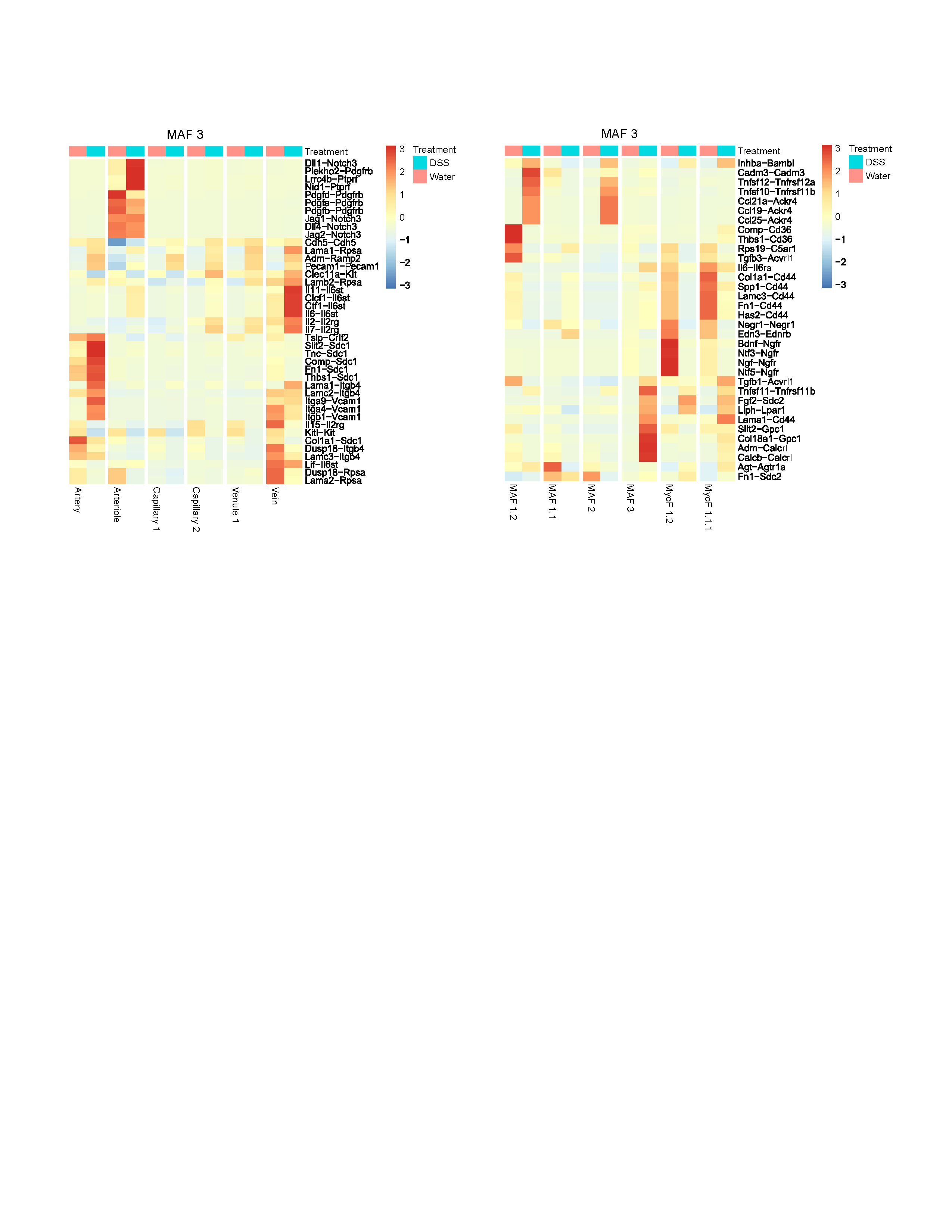

Supplement: S6 Fig — Heatmap of estimated interaction scores based on L–R pair expression between sender cell type MAF3 (IAFs) and receiver cell types: endothelial cell subsets (A) and fibroblast subsets (B). Interaction scores were computed separately for water-treated and DSS-treated mice. Heatmap displays L–R pairs that are differentially expressed between water-treated and DSS-treated mice; for example, ligands that are up-regulated in DSS in MAF3 and receptors that are up-regulated in receiving endothelial subsets (A) or other fibroblast subsets (B). DSS, dextran sulfate sodium; IAF, inflammation-associated fibroblast; L–R, ligand–receptor; MAF, mucosa-associated fibroblast; MyoF, myofibroblast. (TIF) [file pbio.3001532.s006.tif]

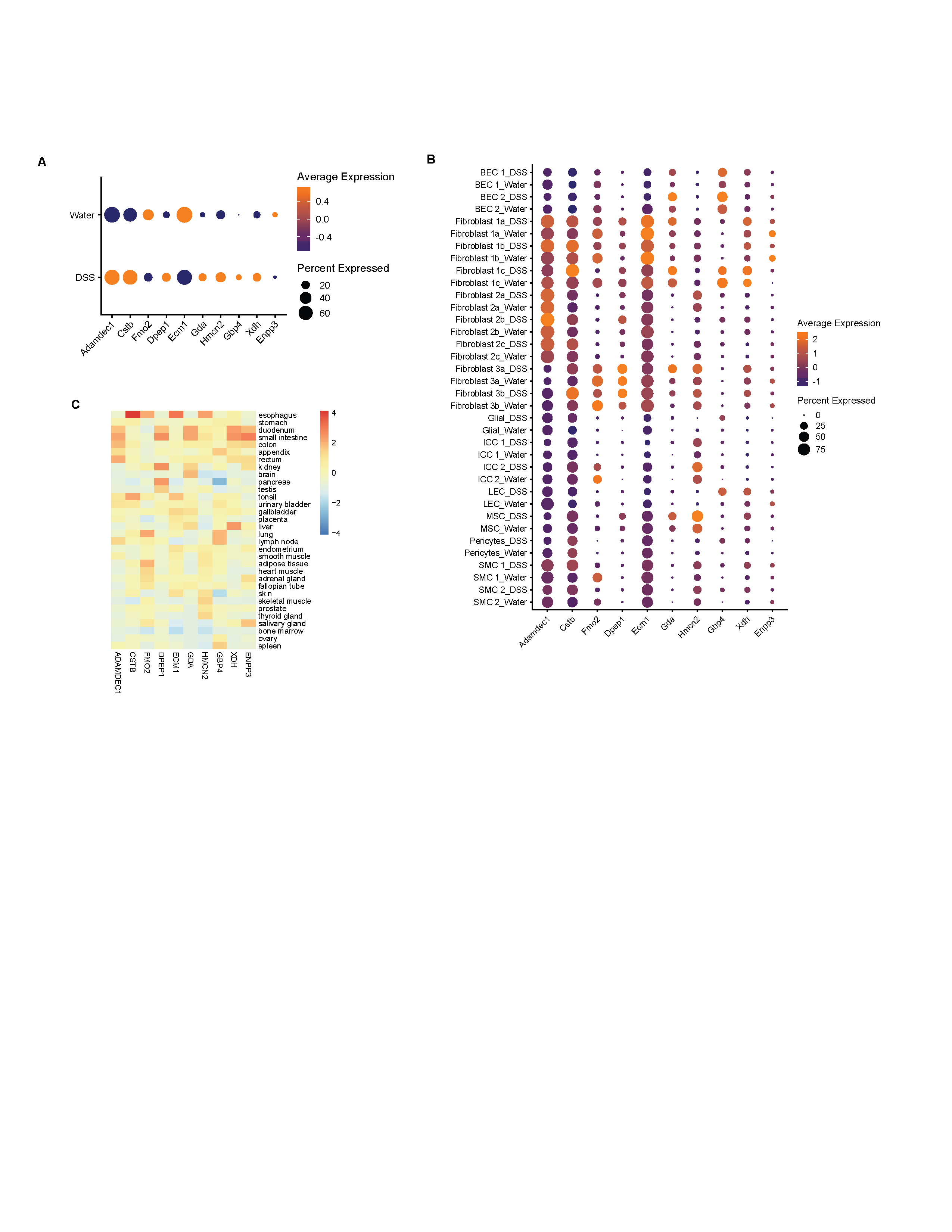

Supplement: S7 Fig — (A) Average expression levels of top 10 genes enriched for expression in the gastrointestinal tissues and also differentially expressed in water- and DSS-treated conditions. Color represents average expression of marker genes across all stromal cell subsets; diameter represents percentage expression of marker genes. (B) Average expression of top 10 genes enriched for expression in the gastrointestinal tract and also differentially expressed in water- and DSS-treated conditions stratified by annotated stromal cell subsets. Color represents average expression of marker gene in each cluster; diameter represents percentage expression of marker gene within cluster. (C) Column scaled average transcript levels of GI tract–enriched genes in several human tissues profiled in the Human Protein Atlas study by Uhlen and colleagues. DEG, differentially expressed gene; DSS, dextran sulfate sodium; GI, gastrointestinal. (TIF) [file pbio.3001532.s007.tif]

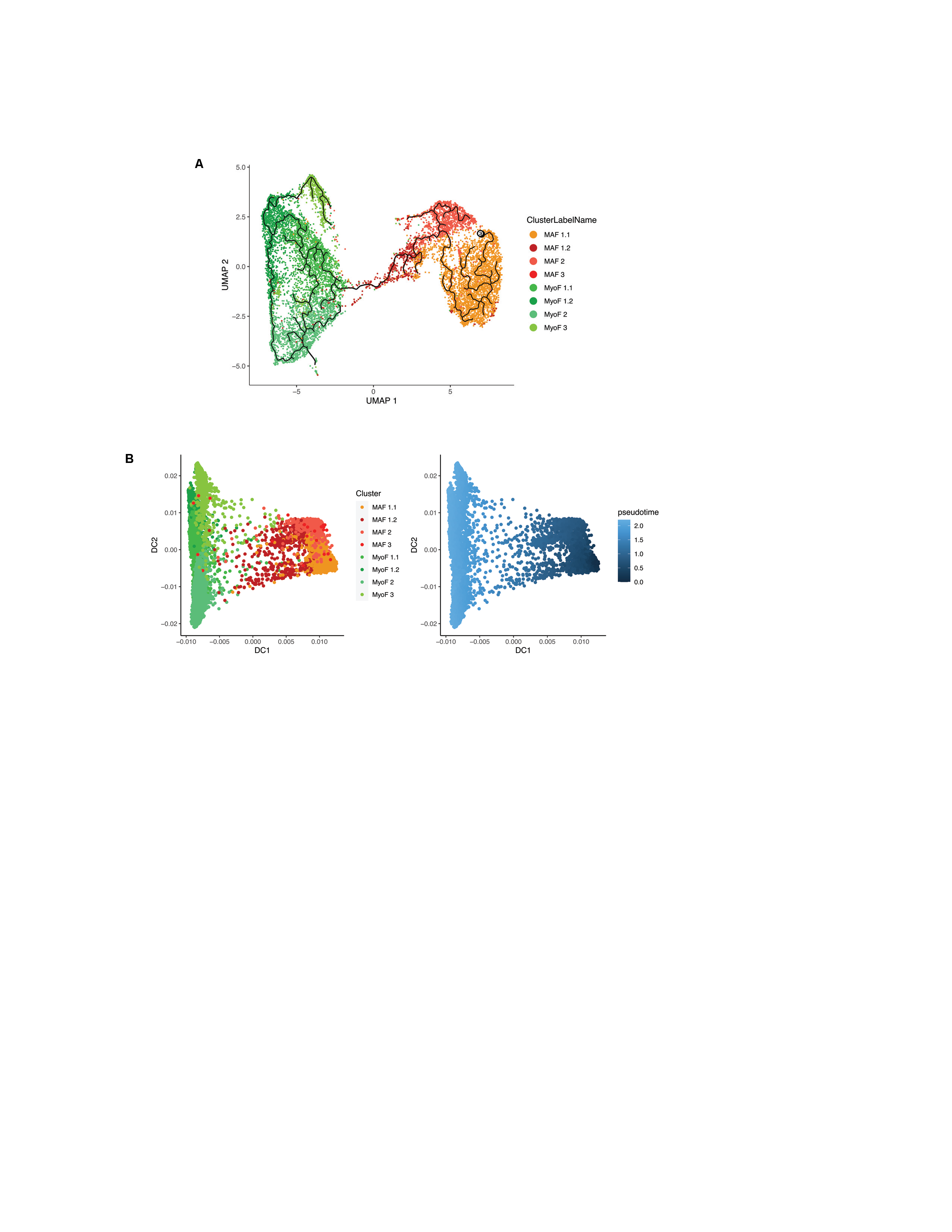

Supplement: S8 Fig — (A) UMAP projections of dimensionality reduction analysis on the subset of MAF and MyoF cells used for the construction of differentiation trajectory overlaid by their lineage subset labels. (B) Visualization of top 2 diffusion components (DC1) from diffusion map analysis of the subset of MAF and MyoF cells overlaid by their lineage subset labels. (C) Diffusion pseudotime reconstruction of the differentiation trajectory from MAFs to MyoFs. MAF, mucosa-associated fibroblast; MyoF, myofibroblast; UMAP, uniform manifold approximation and projection. (TIF) [file pbio.3001532.s008.tif]

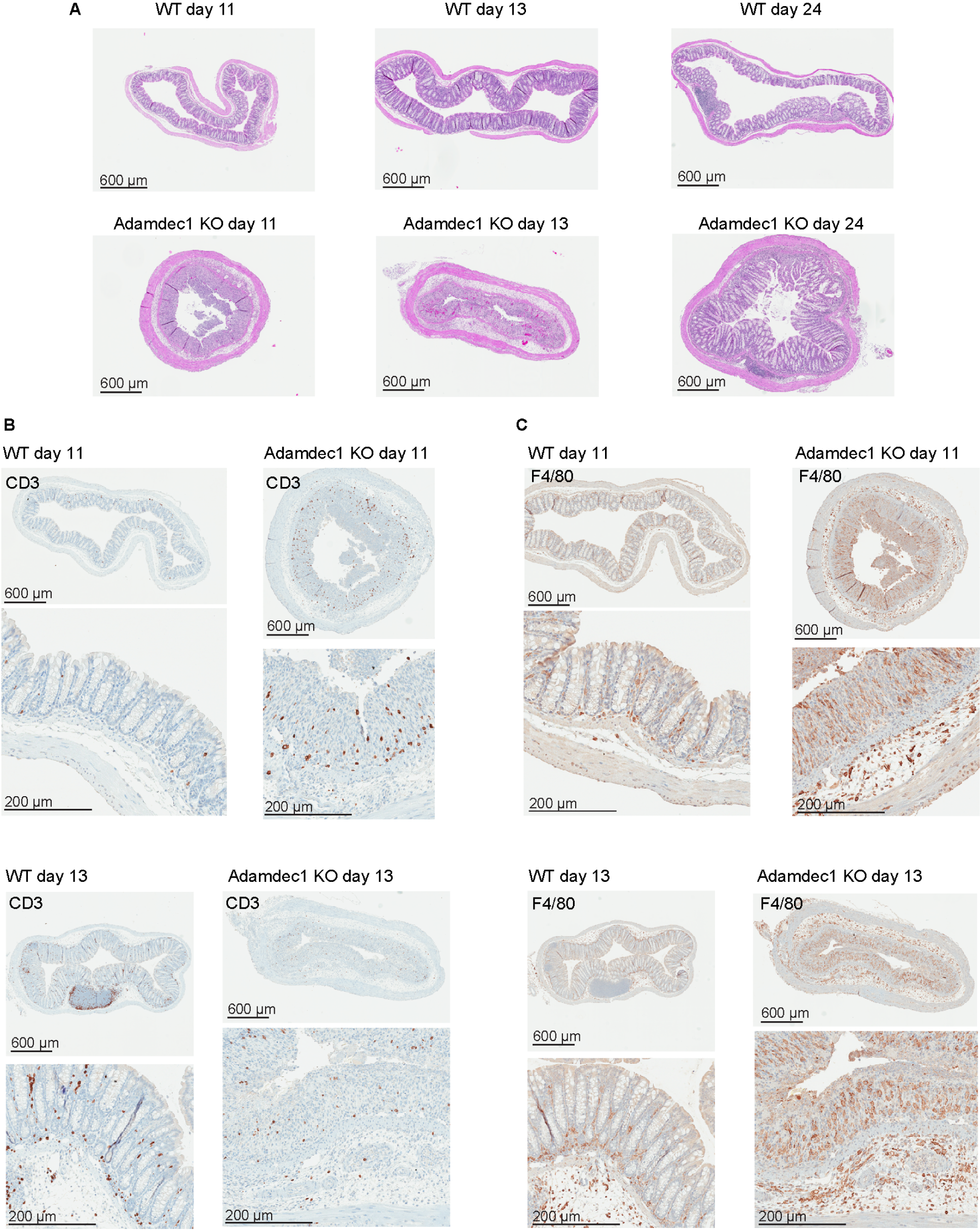

Supplement: S9 Fig — Mice were administered 2% DSS for 7 days, H2O subsequently, and killed on day 11, 13, or 24. HE staining (A) of representative images of colon following DSS in WT and Adamdec1 KO mice. Scale bar, 600 μm. Immunohistochemistry was performed for CD3 (B) and F4/80 (C) on day 11 or 13 after administration of DSS. Scale bars, 600 μm and 200 μm. Representative images, n = 2 per mice per genotype and time point. DSS, dextran sulfate sodium; HE, hematoxylin–eosin; KO, knockout; WT, wild-type. (TIF) [file pbio.3001532.s009.tif]

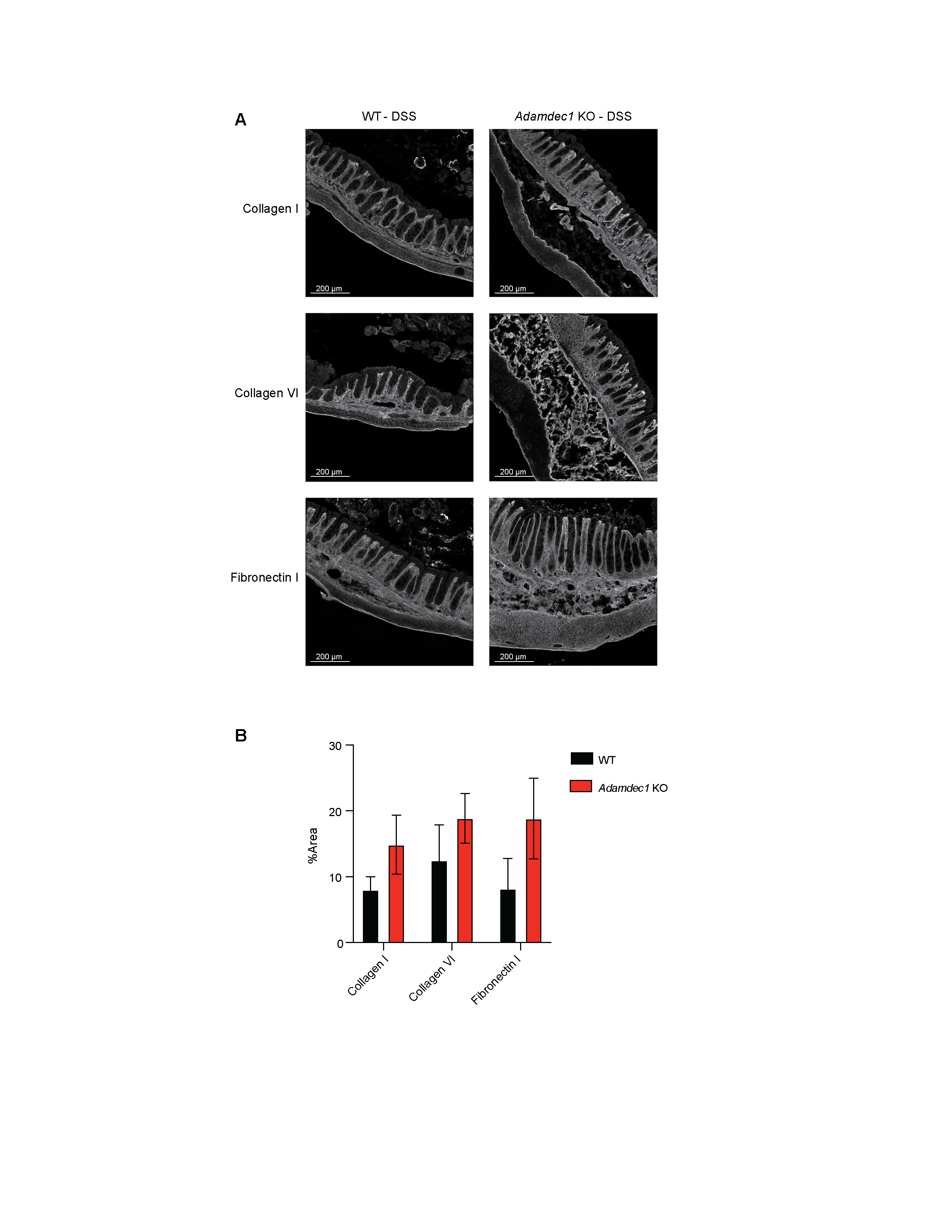

Supplement: S10 Fig — IF imaging was performed on colons from Adamdec1 KO and WT mice with the indicated markers. Mice were administered 2% DSS for 7 days and killed on day 7. (A) Indicated ECM component (white). Scale bar, 200 μm. n = 3 per cohort, raw images representative of 2 experiments. (B) Quantification of indicated ECM components by % area. n = 3 mice per cohort. See S1 Data for source data. DSS, dextran sulfate sodium; ECM, extracellular matrix; IF, immunofluorescence; KO, knockout; WT, wild-type. (TIF) [file pbio.3001532.s010.tif]
